# Exploration of a modified stage for pN0 colon cancer patients

**DOI:** 10.1038/s41598-022-09228-3

**Published:** 2022-03-25

**Authors:** Yunxiao Liu, Hao Zhang, Yuliuming Wang, Mingyu Zheng, Chunlin Wang, Hanqing Hu, Qingchao Tang, Guiyu Wang

**Affiliations:** Department of Colorectal Surgery, the Second Affiliated Hospital of Harbin Medical University, Harbin, 150086 China

**Keywords:** Cancer, Gastroenterology, Oncology

## Abstract

Exploring a modified stage (mStage) for pN0 colon cancer patients. 39,637 pN0 colon cancer patients were collected from the SEER database (2010–2015) (development cohort) and 455 pN0 colon cancer patients from the Second Affiliated Hospital of Harbin Medical University (2011–2015) (validation cohort). The optimal lymph nodes examined (LNE) stratification for cancer-specific survival (CSS) was obtained by X-tile software in the development cohort. LNE is combined with conventional T stage to form the mStage. The novel N stage was built based on the LNE (N0a: LNE ≥ 26, N0b: LNE = 11–25 and N0c: LNE ≤ 10). The mStage include mStageA (T1N0a, T1N0b, T1N0c and T2N0a), mStageB (T2N0b, T2N0c and T3N0a), mStageC (T3N0b), mStageD (T3N0c, T4aN0a and T4bN0a), mStageE (T4aN0b and T4bN0b) and mStageF (T4aN0c and T4bN0c). Cox regression model showed that mStage was an independent prognostic factor. AUC showed that the predictive accuracy of mStage was better than the conventional T stage for 5-year CSS in the development (0.700 vs. 0.678, *P* < 0.001) and validation cohort (0.649 vs. 0.603, *P* = 0.018). The C-index also showed that mStage had a superior model-fitting. Besides, calibration curves for 3-year and 5-year CSS revealed good consistencies between observed and predicted survival rates. For pN0 colon cancer patients, mStage might be superior to conventional T stage in predicting the prognosis.

## Introduction

Globally, colon cancer (CC) is one of the most common cancers worldwide and the major causes of cancer-related mortality^1,2^. For resectable CC, surgery combined with systematic lymph node dissection is considered as the primary treatment^3^. Although many prognostic markers have been identified to date, tumor stage is the most widely used prognostic factor^4^. The American Joint Commission on Cancer (AJCC) tumor-node-metastasis (TNM) classification, which is based on the depth of tumor invasion of the intestinal wall and the number of positive lymph nodes, is the most important factor in determining prognosis and subsequent therapeutic methods.

In recent years, the number of lymph nodes examined (LNE) for pN0 CC patients has attracted substantial attention due to its unique prognostic value^5^. Studies have shown that the greater the number of LNE, the better the disease-free survival (DFS) and overall survival (OS), especially in pN0 patients^6–8^. LNE is an independent risk factor for survival in patients with CC. Moreover, the LNE is an important indicator to ensure accurate staging of lymph nodes because it helps to assess the extent of lymph node involvement^9,10^. The National Comprehensive Cancer Network (NCCN) guidelines recommend that at least 12 lymph nodes need to be dissected intraoperatively for CC patients to effectively assess postoperative pathological staging^11^. In recent clinical practice, about 30–50% of CC patients still have inadequate lymph node dissection^12,13^.

However, the prognostic stratification for CC patients with negative node metastasis diseases has been only determined by T stage, regardless of the nodal information. In other words, the conventional staging system might be inappropriate for pN0 patients and the number of LNE could be taken into consideration to better stratify patients with different prognosis. Therefore, this study used data from the SEER database to determine the optimal stratification of LNE for pN0 CC patients and subsequently, construct a modified stage (mStage) for this special population based on conventional T stage and novel N stage (nN stage). In addition, our departmental data was used to further validated the capability of the mStage.

## Methods

### Patients

CC cases were collected from the SEER database between January 2010 and December 2015, and treatment data were acquired from SEER custom data via further application.

Inclusion criteria included: (1) The pathological diagnosis was CC without positive lymph nodes and distant metastasis; (2) aged ≥ 18 years old; (3) patients with complete records of cancer-specific survival months and vital status; (4) CC was the only primary malignancy. Exclusion criteria included: (1) patient received neoadjuvant and adjuvant therapy; (2) patients without complete follow-up data; (3) the basic information of the patient is incomplete.

In addition, 445 CC cases from the Second Affiliated Hospital of Harbin Medical University between January 2011 and December 2015 were also enrolled in this research as a validation cohort. The last follow-up was in October 2021. Inclusion and exclusion criteria for validation cohort were the same as those for development cohort (SEER).

### Statistical analysis

All the statistical analyses were calculated in statistical software package SPSS 22.0 (IBM Corp, Armonk, NY, USA) and R software (version 3.6.1 https://www.r-proje ct.org/). The clinical characteristics of patients were summarized by number and percentage. In order to obtain the new N stage, the most appropriate cut-off value of LNE for CSS were obtained by X-tile software (version 3.6.1 https://medicine.yale.edu/lab/rimm/research/software/). Cox proportional hazard regression was applied to investigate the relationship between mStage and CSS. Concordance index (C-index) and receiver operating characteristic (ROC) curve were used to determine the efficiency of mStage. Kaplan–Meier curves were generated and analyzed using log-rank tests. The difference was considered statistically significant for a two-sided P < 0.05.

## Result

### Patient characteristics

According to the screening criteria, 39,637 patients from the SEER database (development cohort) and 455 patients from the Chinese population (validation cohort) were identified in this study. In the development cohort, female (51.0%), older than 65 years (65.0%), accounted for a higher proportion of patients, while male (60.5%), less than 65 years (54.5%), accounted for a higher proportion of patients in the validation cohort. In all patients, most proportions were found in right colon (64.2% and 50.5%), adenocarcinoma (92.5% and 77.4%), grade I/II (87.8% and 89.75%). The mean number of LNE in the development and validation cohorts was 18.98 ± 9.52 and 16.94 ± 7.77, respectively. The detailed data was summarized in Table 1.

**Table 1:** Characteristics of patients in the development and validation cohorts.

Characteristics	Development cohort (n = 39,637)	Validation cohort (n = 455)
**Race, n (%)**		
White	31,528 (79.5)	0 (0.0)
Black	4452 (11.2)	0 (0.0)
Other	3657 (9.3)	455(100)
**Gender, n (%)**		
Male	19,437 (49.0)	275(60.5)
Female	20,200 (51.0)	180(39.5)
**Grade, n (%)**		
Grade I/II	34,807 (87.8)	570 (89.7)
Grade III/IV	4830(12.2)	47 (10.3)
**Histological type, n (%)**		
Adenocarcinoma	36,685 (92.5)	352 (77.4)
Mucinous/signet ring-cell carcinoma	2952 (7.5)	103(22.6)
**Age (years), n (%)**		
< 65	13,845 (35.0)	248 (54.5)
≥ 65	25,783(65.0)	207(45.5)
**Tumor location, n (%)**		
Right colon	25,451 (64.2)	230(50.5)
Left colon	14,186 (35.8)	225(49.5)
**T stage, n (%)**		
T1	8593 (21.7)	75(16.5)
T2	9121 (23.0)	92(20.2)
T3	19,447 (49.0)	112 (24.6)
T4a	1535 (3.9)	128 (28.1)
T4b	941 (2.4)	48(10.5)
**Number of LNE**	18.98 ± 9.52	16.94 ± 7.77

### Construction of the modified TNM stage

**Figure 1:**
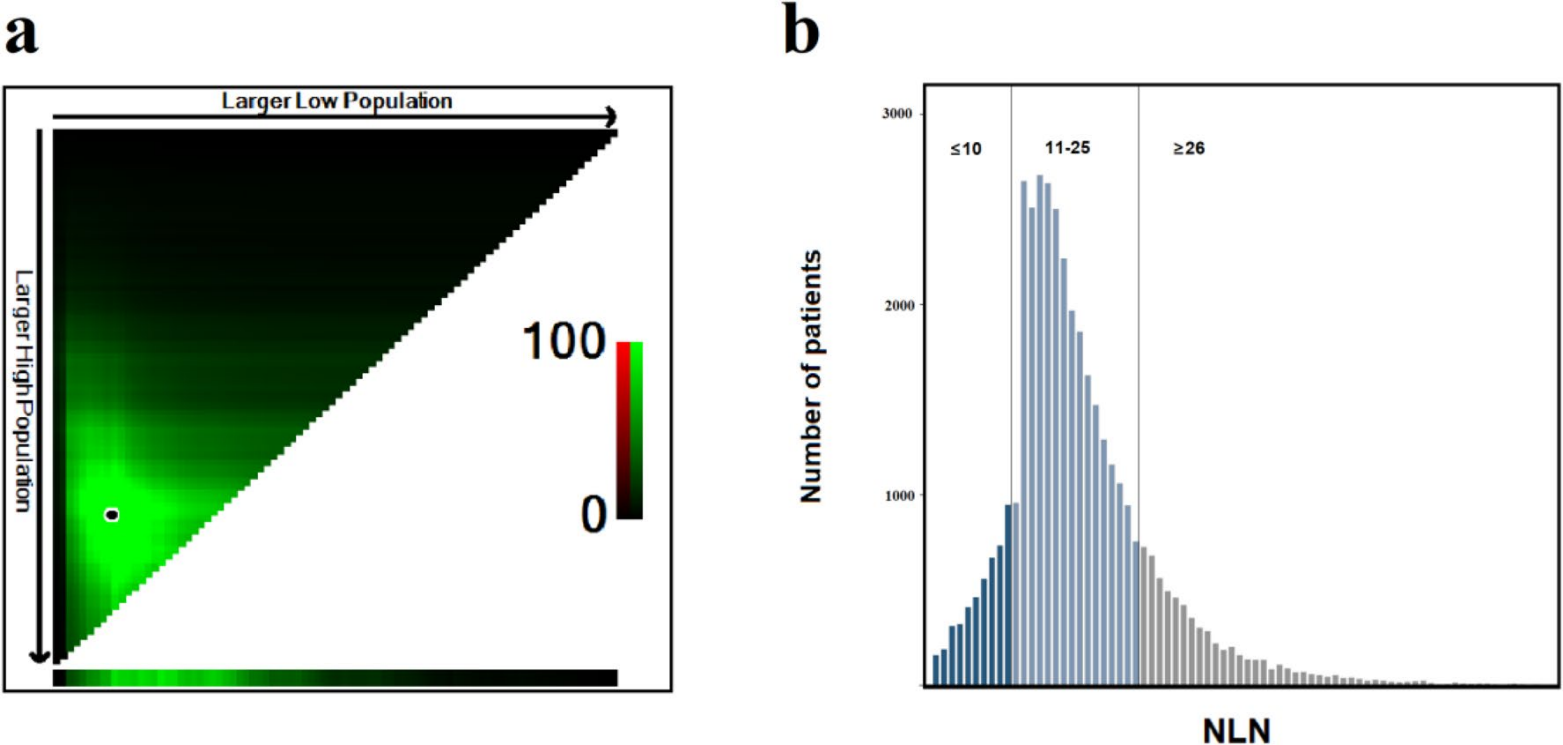
X-tile analysis of CSS in the development cohort. a X-tile plot. b histogram plot.

**Figure 2:**
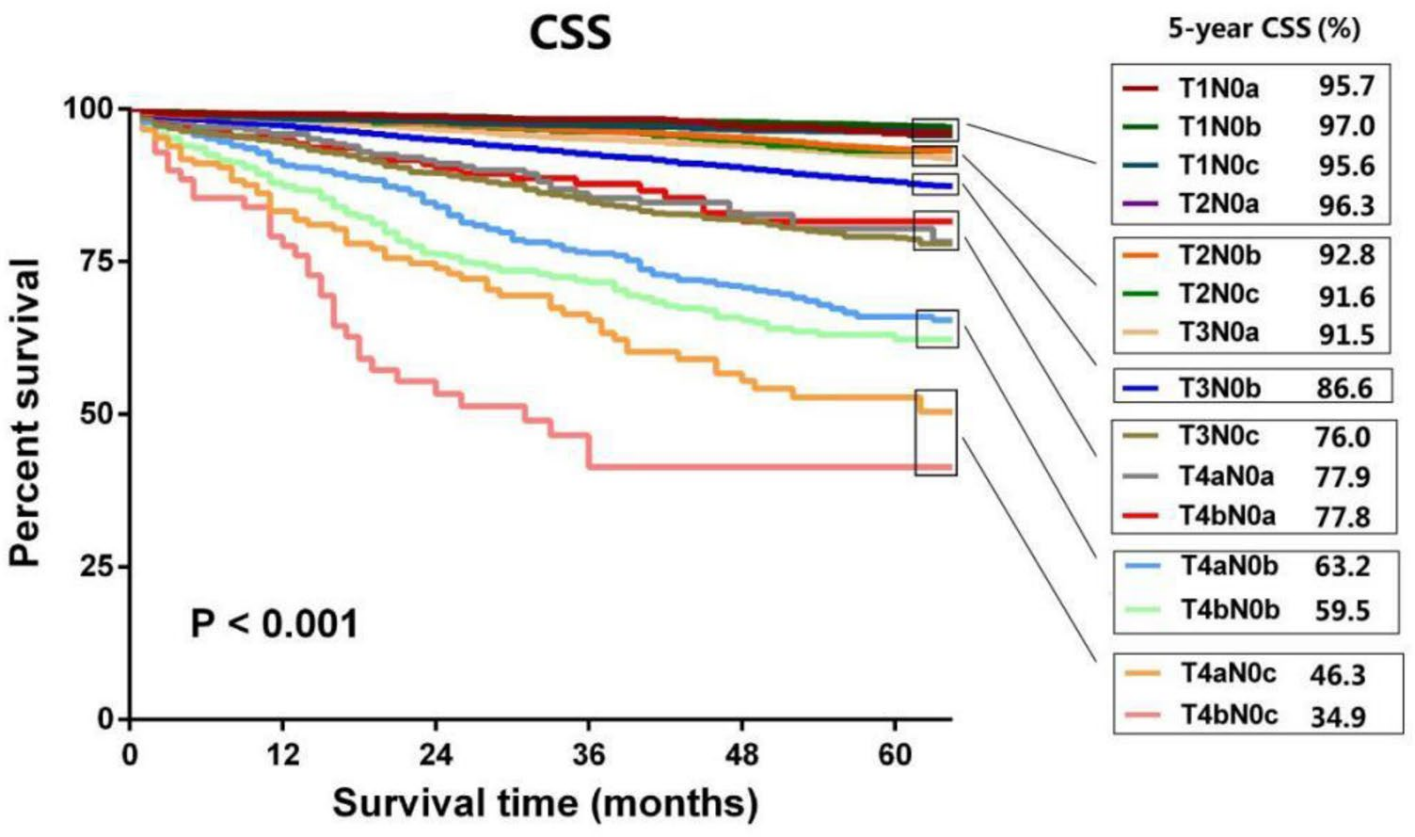
Kaplan–Meier curves for patients in different subgroups in the development cohort.

**Figure 3:**
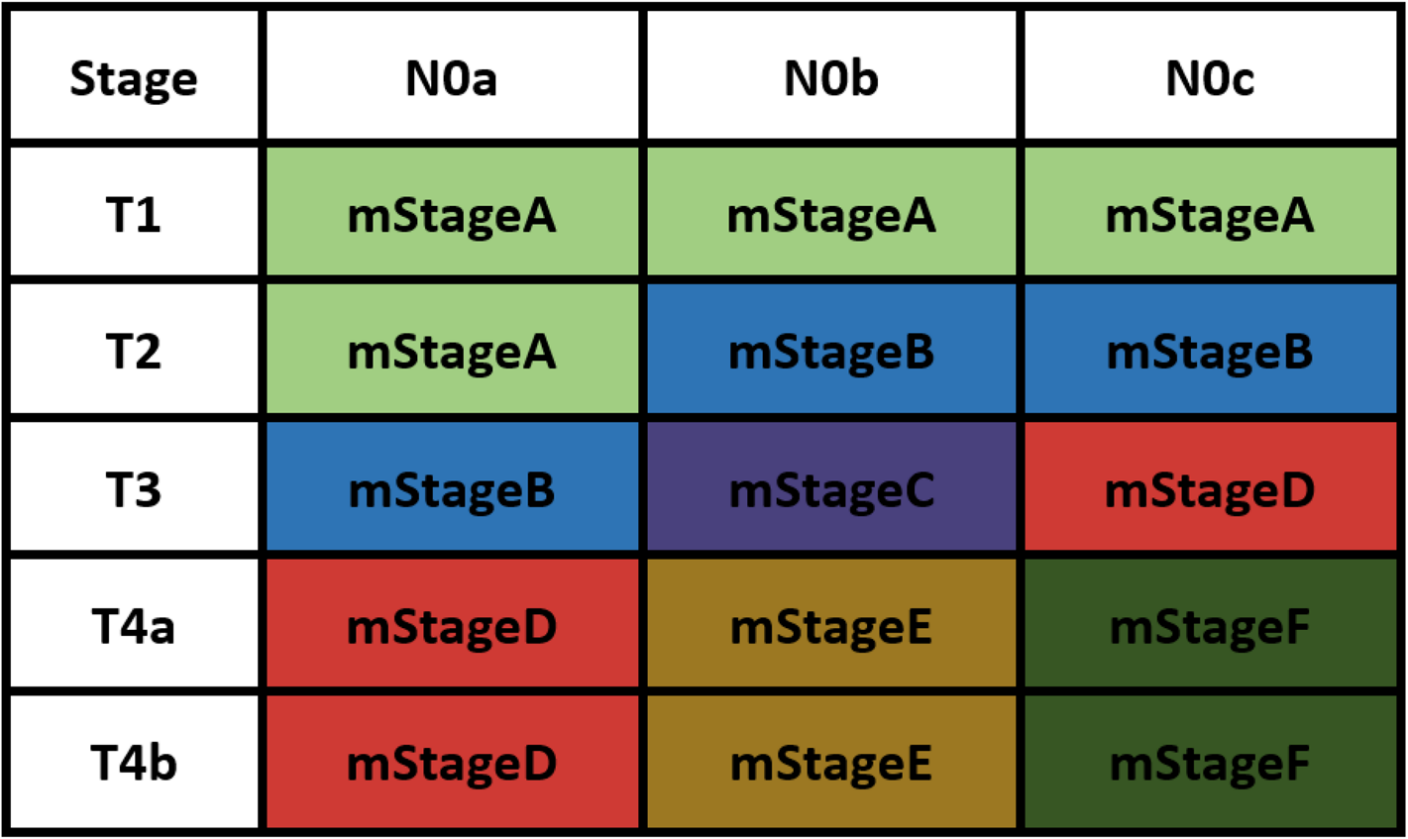
Modified TNM staging system.

In the development cohort, the optimal stratification of LNE for CSS was achieved by the X-tile software and was applied to build the novel N stage (N0a: LNE ≥ 26, N0b: LNE = 11–25 and N0c: LNE ≤ 10) (Table 2) (Fig. 1a,b). Kaplan–Meier survival analysis results showed that there were significant differences in prognosis among the three LNE groups (*P* < 0.001) (Suppl. Fig. S1). Then, patients were redivided into 15 subgroups by combining the conventional T stage (T1, T2, T3, T4a and T4b) with the nN stage (N0a, N0b and N0c) and the prognosis of these subgroups were further compared (Table 3; Fig. 2). Using T1N0a as a reference, all subgroups were redivided into six modified stages (mStage) based on the 5-year CSS rates and HRs. The mStage include mStageA (T1N0a, T1N0b, T1N0c and T2N0a), mStageB (T2N0b, T2N0c and T3N0a), mStageC (T3N0b), mStageD (T3N0c, T4aN0a and T4bN0a), mStageE (T4aN0b and T4bN0b) and mStageF (T4aN0c, and T4bN0c) (Fig. 3). The 5-year CSS rates for mStageA, B, C, D, E and F were 96.5%, 92.3%, 86.6%, 76.4%, 61.8% and 40.9%, respectively (*P* < 0.001).

**Table 2:** Grouping of nN stage.

Novel N stage	LNE
N0a	26 +
N0b	11–25
N0c	1–10

**Table 3:** Survival analysis among different subgroups.

Group	5-year CSS rate (%)	HR	95%CI	*P*
T1N0a	95.7	1		
T1N0b	97.0	0.851	0.563–1.288	0.446
T1N0c	95.6	1.317	0.844–2.055	0.225
T2N0a	96.3	0.994	0.613–1.612	0.980
T2N0b	92.8	1.871	1.263–2.771	0.002
T2N0c	91.6	2.373	1.522–3.699	0.000
T3N0a	91.5	2.345	1.577–3.486	0.000
T3N0b	86.6	3.865	2.641–5.658	0.000
T3N0c	76.0	7.954	5.366–11.792	0.000
T4aN0a	77.9	6.844	4.255–11.007	0.000
T4aN0b	63.2	12.216	8.230–18.132	0.000
T4aN0c	46.3	22.456	14.506–34.762	0.000
T4bN0a	77.8	7.229	4.347–12.022	0.000
T4bN0b	59.5	14.732	9.861–22.009	0.000
T4bN0c	34.9	35.416	22.136–56.662	0.000

### Superiority of the modified TNM staging system

**Figure 4:**
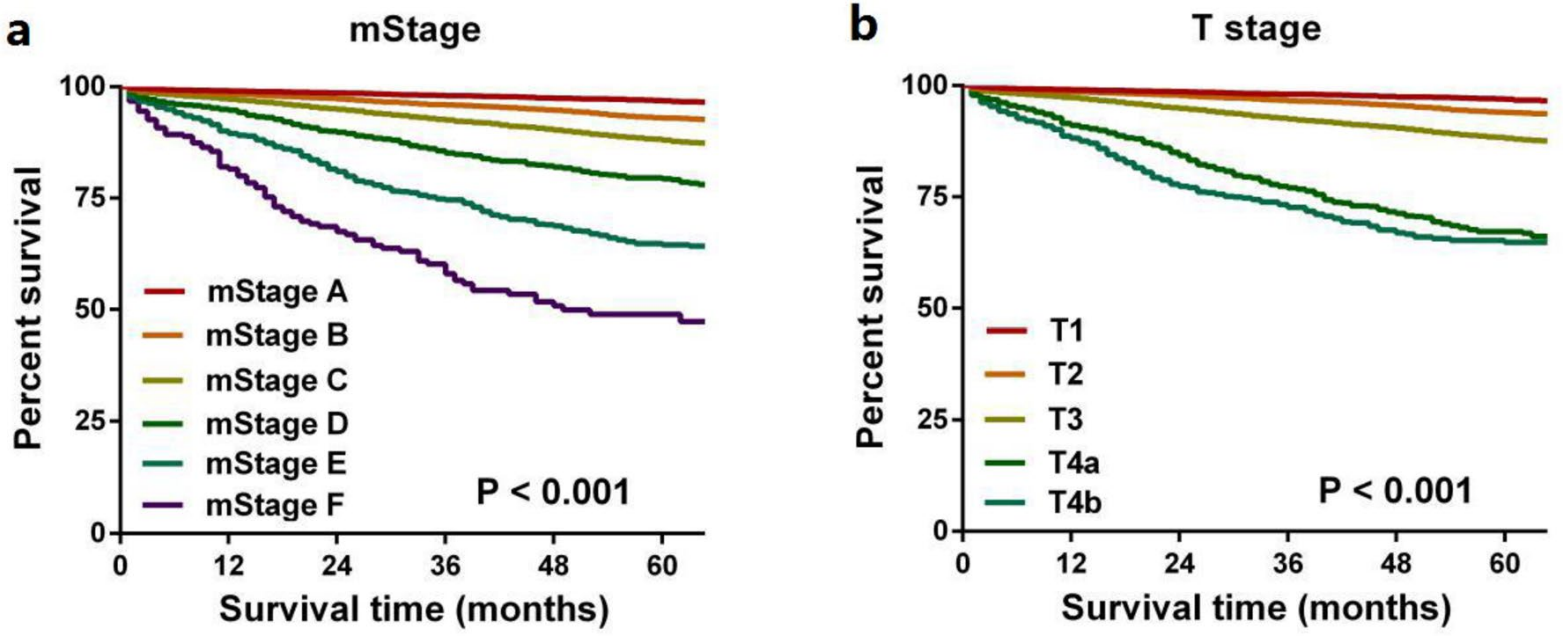
Kaplan–Meier curves stratified by mStage (**a**) and conventional TNM stage (**b**) in the development cohort.

**Figure 5:**
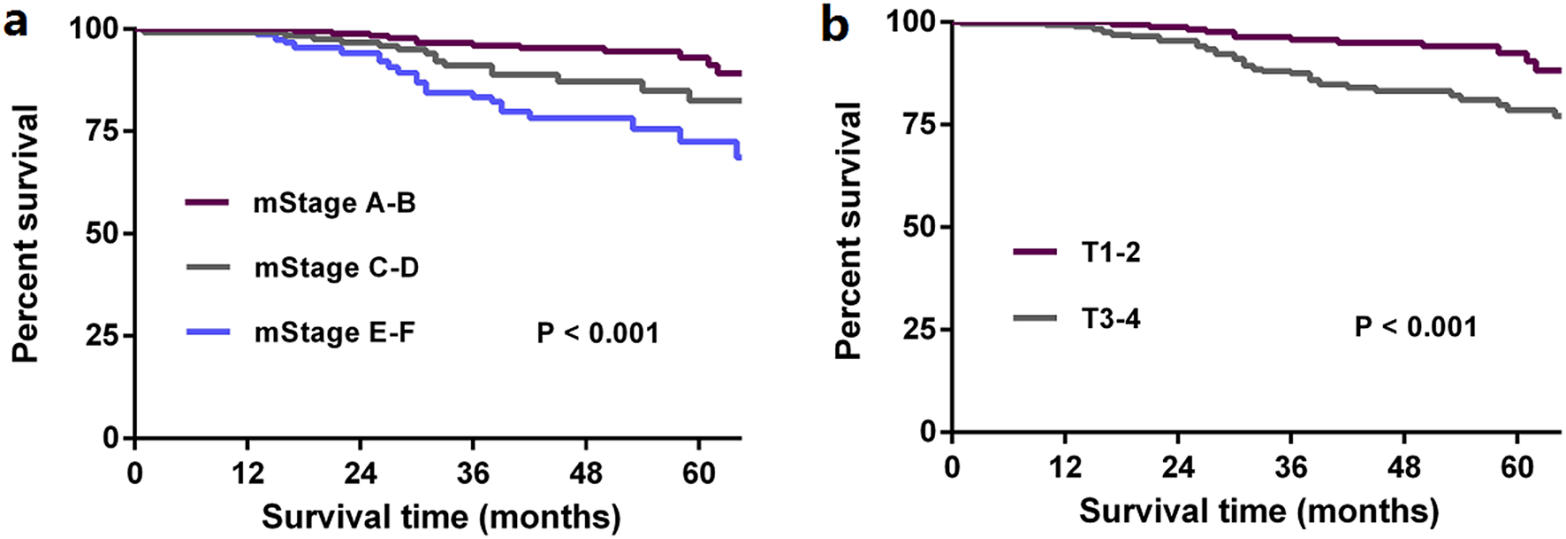
Kaplan–Meier curves stratified by mStage (**a**) and conventional TNM stage (**b**) in the validation cohort.

Cox proportional hazard regression model showed that mStage was still an independent prognostic factor of CSS after eliminating confounding factors (Table 4). In addition, mStage was also found to be an independent prognostic factor for OS and CSS excluding those died from other causes (Suppl. Table S1), 2). Figures 4a,b and 5a,b show survival curves stratified by conventional TNM stage and mStage and prognostic stratification using the mStage is much clearer than with conventional TNM stage in the development and validation cohorts.

**Table 4:** Cox regression analyses of factors related to CSS in the development cohort.

Characteristics	Univariate analysis		Multivariable analysis	
	HR [95%CI]	*P*	HR [95%CI]	*P*
**Race**				
White	1		1	
Black	1.104 [0.994–1.226]	0.063	1.284 [1.156–1.427]	0.000
Other	0.769 [0.671–0.880]	0.000	0.814 [0.710–0.932]	0.003
**Gender**				
Male	1		1	
Female	1.073 [1.001–1.149]	0.045	0.966 [0.901–1.035]	0.323
**Grade**				
I + II	1		1	
III + IV	1.659 [1.517–1.813]	0.000	1.260 [1.151–1.379]	0.000
**Histological type**				
Adenocarcinoma	1		1	
Mucinous/signet-cell carcinoma	1.152 [1.017–1.304]	0.026	0.876 [0.773–0.992]	0.038
**Age (years)**				
< 65	1		1	
≥ 65	2.693 [2.461–2.947]	0.000	2.293 [2.093–2.512]	0.000
**Tumor location**				
Right colon	1			
Left colon	0.985 [0.917–1.058]	0.677		
**mStage**				
A	1		1	
B	2.138 [1.855–2.464]	0.000	2.008 [1.741–2.315]	0.000
C	3.961 [3.476–4.515]	0.000	3.562 [3.123–4.063]	0.000
D	7.928 [6.783–9.266]	0.000	6.875 [5.877–8.041]	0.000
E	13.488 [11.621–15.654]	0.000	11.548 [9.931–13.428]	0.000
F	26.761 [21.681–33.031]	0.000	23.718 [19.196–29.306]	0.000

**Figure 6:**
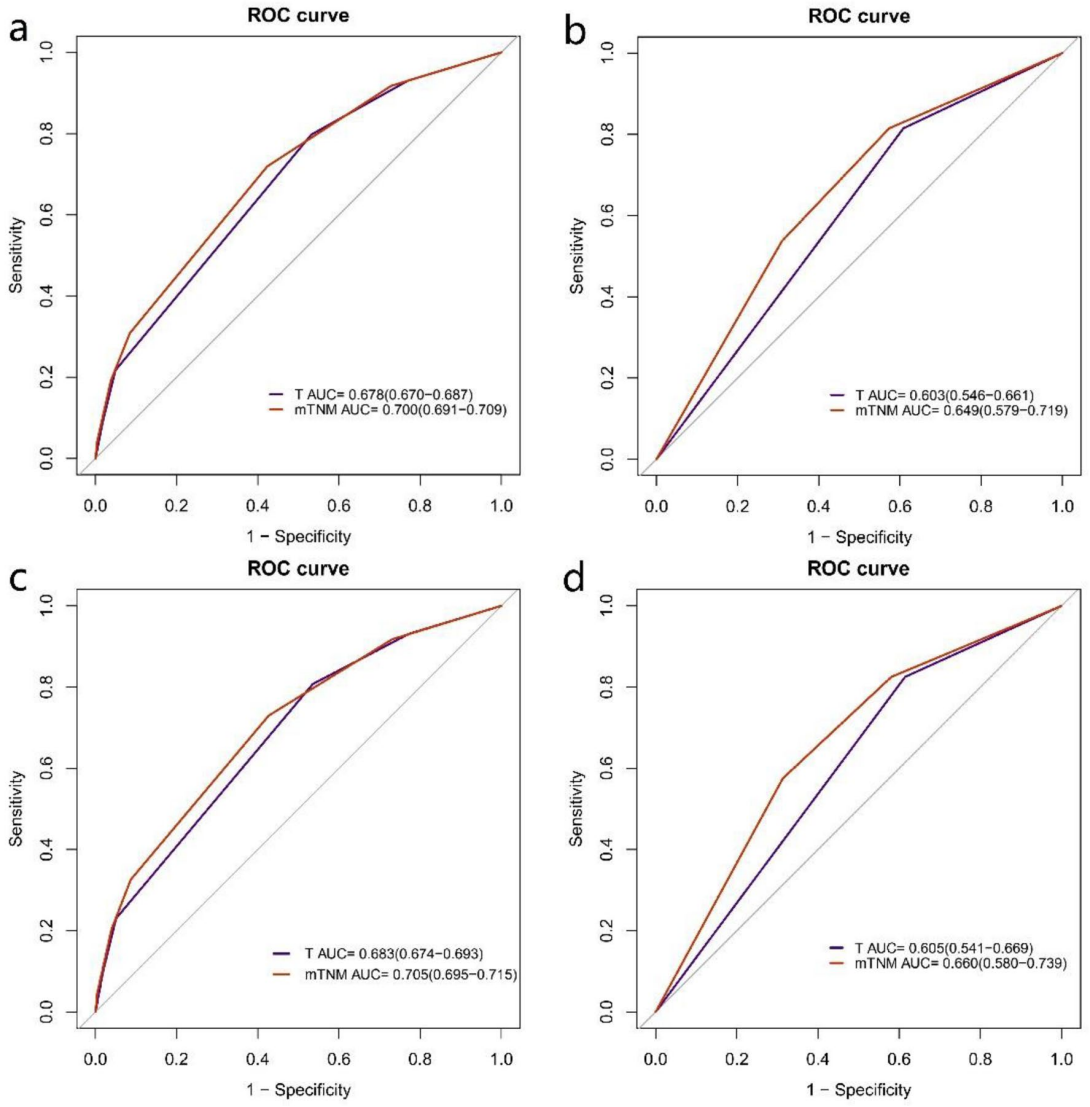
The AUCs of the mStage and conventional TNM stage. (**a**) Comparison of the 5-year AUCs in the development cohort. (**b**) Comparison of the 5-year AUCs in the validation cohort. (**c**) Comparison of the 3-year AUCs in the development cohort. (**d**) Comparison of the 3-year AUCs in the validation cohort.

In the development cohort, the C-indices of the mStage and conventional TNM stage were 0.699 (95%CI = 0.695–0704) and 0.678 (95%CI = 0.674–0.682) (P < 0.001), respectively, also indicating the better discrimination ability of the mStage compared with conventional TNM stage. The AUCs of the mStage and TNM stage at 5-year were 0.700 (95%CI = 0.691–0709) and 0.678 (95%CI = 0.670–0687) (P < 0.001) (Fig. 6a), respectively.

In validation cohort, the C-indices of the mStage and conventional TNM stage were 0.644 (95%CI = 0.632–0.697) and 0.613 (95%CI = 0.587–0.640) (P < 0.001) and the AUCs of the mStage and TNM stage at 5-year were 0.649 (95%CI = 0.579–0.719) and 0.603 (95%CI = 0.546–0.661), respectively (p = 0.018) (Fig. 6b).

In addition, AUCs of the mStage and TNM stage at 3-year were drawn based on the new staging also indicating the better discrimination ability of the mStage in the development and validation cohort (Fig. 6c,d).

**Figure 7:**
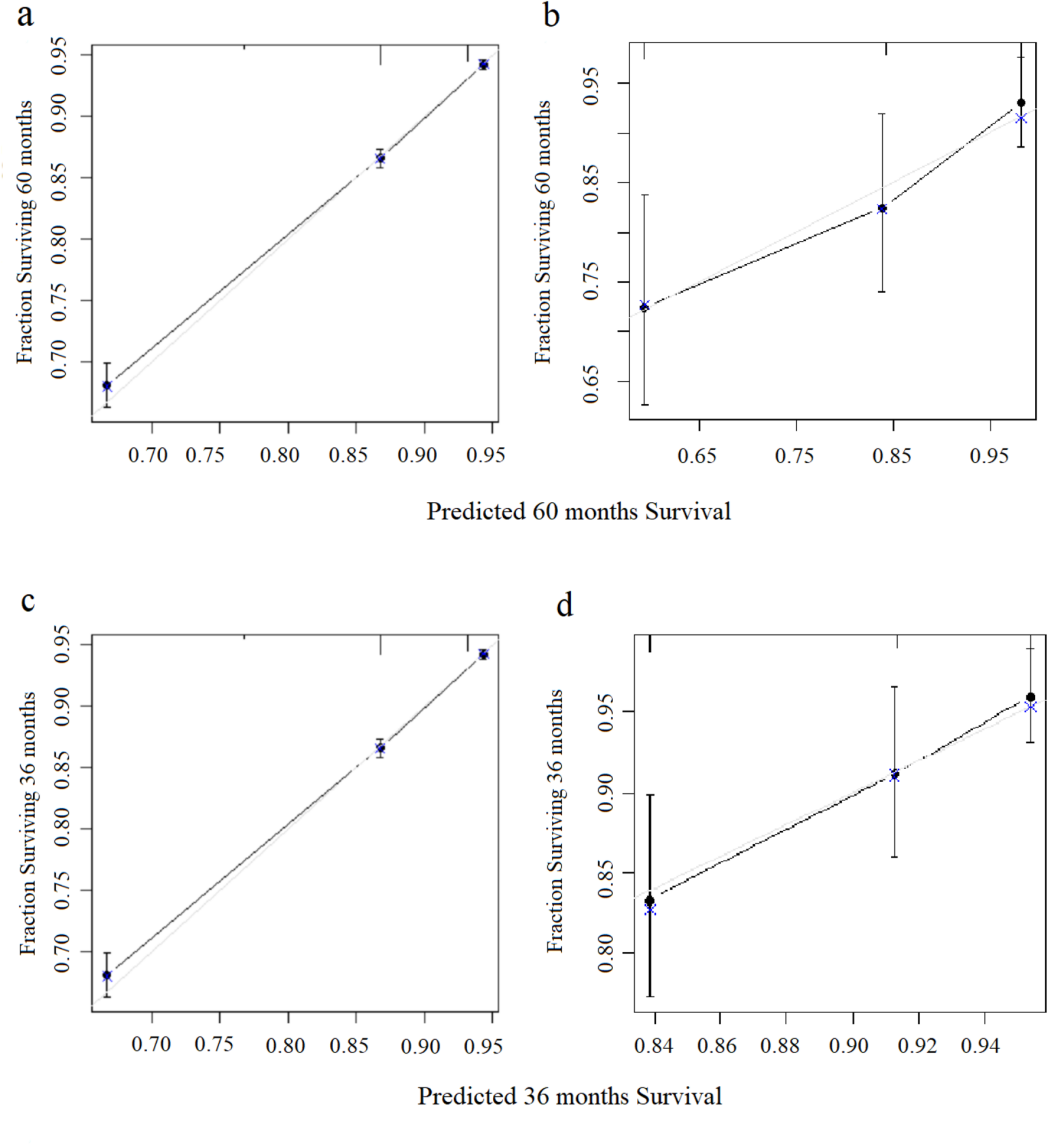
The calibration curves of the mStage. (**a**) Calibration curves for 5-year CSS in the development cohort. (**b**) Calibration curves for 5-year CSS in the validation cohort. (**c**) Calibration curves for 3-year CSS in the development cohort. (**d**) Calibration curves for 3-year CSS in the validation cohort.

What’ s more, the calibration curves for 3-year and 5-year CSS also showed a satisfactory predictive accuracy in the development and validation cohorts (Fig. 7a–d).

## Discussion

Nowadays, CC is associated with a higher incidence of gastrointestinal cancers and poses a major public health challenge due to its high mortality rate^1^. The AJCC TNM staging system is the most widely applied system in clinical practice to evaluate the survival status, treatment and prognosis of patients. Among them, N stage was divided mainly according to whether there was lymph node metastasis or the number of positive lymph nodes: N0 (no metastatic LNE), N1 (N1a: 1 metastatic LNE; N1b: 2–3 metastatic LNE; N1c: cancer nodule formation) and N2 (N2a: 4–6 metastatic LNE; N2a ≥ 7 metastatic LNE). It can be seen that there is no further stratification in N0 stage. Hence, pN0 stage patients were only stratified according to the T stage, remains a controversial issue.

At present, the number of LNE has been shown to be an independent prognostic factor in multiple cancer types, especially in CC. Higher LNE has been associated with improved survival of pN0 CC patients but the mechanism of the relationship between the two is unclear^6,9,14^. Several hypotheses have been proposed. One possible reason is that the greater the number of LNE is associated with a greater chance of a positive node being examined and a more accurate tumor stage^15,16^. Assessing the number of LNE helps with reducing the likelihood of misclassifying stage III disease as stage I or II and improve prognosis, particularly for pN0 CC patients^17–19^. In addition, an increase in the number of LNE may be an indicator of better treatment, including complete tumor resection and adequate pathological evaluation. Another explanation is that the increase in the number of negative lymph nodes indicates a stronger immune response. Once the immune system detects the presence of tumor cells, local lymph nodes will increase, and more lymph nodes will be easier to be examined in postoperative pathology. Studies have found that LNE are correlated with local neutrophil and lymphocyte infiltration by analyzing the tumor microenvironment^5^. All the above studies proved the relationship between LNE and prognosis through data analysis, but did not specify the optimal stratification of LNE in pN0 CC patients. In this study, the optimal stratification of LNE for CSS was achieved by the X-tile software (N0a: LNE ≥ 26, N0b: LNE = 11–25 and N0c: LNE ≤ 10) and the Kaplan–Meier survival analysis results showed that there were significant differences in prognosis among the three LNE groups (P < 0.001) that proves that our results are meaningful.

The AJCC 8th TNM classification system recommends a minimum of 12 lymph nodes to effectively assess patient survival benefits. The number of LNE can be used effectively as a marker of surgical and pathological adequacy. But LNE are often influenced by tumor location, tumor size and patient age, and especially by the skill of the surgeon and the diligence of the pathologist^12,20–22^. When the number of LNE is insufficient, the conventional TNM system is used for staging, and patients may be misjudged, especially for those determined as N0 stage cases. The inclusion of the number of LNE in the modified staging system could better stratify patients compared with conventional method to some extent.

In addition, there is a great deal of debate about the number of LNE at least 12. Ning et al. found that the optimal cut-off value of LNE should be 18 in pN0 CC patients^23^.Therefore, the cut-off value of the number of LNE is still controversial. We urgently need a new and convincing staging system for clinical use.

In this study, the optimal stratification of LNE was achieved by the X-tile software (nN stage: (N0a: LNE ≥ 26, N0b: LNE = 11–25 and N0c: LNE ≤ 10) and there were significant statistical differences between the three groups. Subsequently, a modified TNM stage was constructed based on conventional T stage and nN stage. To make the new system more rational in distinguishing patients with different outcomes, all patients were unified into six modified stages (mStage) according to the HRs and survival curves. The KM CSS curves show that the mStage can better classify patients with similar prognosis than the conventional stage. In addition, the AUC and C-index of mStage were significantly higher than those of conventional TNM staging system in both development and validation cohorts, indicating that the mStage has potential advantages over conventional stage in predicting survival.

There are several innovations in our research. First of all, the selection of LNE cut-off value took into account the patients with insufficient LNE, making the nN stage system more universal. Then, we further analyzed the prognostic interaction between nN stage and conventional T stage and constructed a modified staging system for pN0 CC patients, which showed superior predictive power compared with conventional TNM staging system. Finally, we did validation cohort to make our results more convincing.

This study has several limitations. Firstly, we proposed stratification of LNE for the first time, while there was no consensus on stratification results, which may limit the application and promotion of the mStage system. Secondly, this study is a retrospective analysis, which needs to be further verified by some prospective clinical studies. Thirdly, the sample size of the validation cohort seems to be insufficient, requiring a larger sample analysis to verify the accuracy of the modified staging system in the future.

In conclusion, the mStage system could predict the prognosis of pN0 CC patients and showed superior predictive power compared with conventional TNM staging system.

### Ethical approval

This study received ethical approval from the Second Affiliated Hospital of Harbin Medical University. The study used de-identified data and adhered to World Medical Association’s Declaration of Helsinki for Ethical Human Research. SEER is a publicly available database with anonymized data; no ethical review was required.

### Informed consent

Informed consent has been obtained from 455 colorectal cancer patients and their families.

## Supplementary Information

Supplementary Information.

## Data Availability

The study data of development cohort are available from the SEER database (user ID: 14,262-Nov2019, https://seer.cancer.gov/). The study data of validation cohort used and/or analyzed during the current study are available from the Second Affiliated Hospital of Harbin Medical University, China.
