# Supplementary material for: Exploration of a modified stage for pN0 colon cancer patients
Source: Sci Rep. 2022 Mar 25;12:5214. doi: 10.1038/s41598-022-09228-3 (PMC8956689; doi:10.1038/s41598-022-09228-3)

| **Characteristics** | **Univariate analysis** | | **Multivariable analysis** | |
| --- | --- | --- | --- | --- |
|  | **HR [95%CI]** | **P** | **HR [95%CI]** | **P** |
| **Race** |  |  |  |  |
| White | 1 |  | 1 |  |
| Black | 0.949 [0.884-1.018] | 0.142 | 1.133 [1.056-1.216] | 0.001 |
| Other | 0.663[0.671-0.726] | 0.000 | 0.716 [0.653-0.784] | 0.003 |
| **Gender** |  |  |  |  |
| Male | 1 |  |  |  |
| Female | 0.967[0.925-1.010] | 0.127 |  |  |
| **Grade** |  |  |  |  |
| I+II | 1 |  | 1 |  |
| III+IV | 1.397 [1.315-1.483] | 0.000 | 1.138 [1.071-1.210] | 0.000 |
| **Histological type** |  |  |  |  |
| Adenocarcinoma | 1 |  | 1 |  |
| Mucinous/signet-cell carcinoma | 1.281 [1.187-1.381 | 0.000 | 1.052 [0.974-1.136] | 0.194 |
| **Age (years)** |  |  |  |  |
| < 65 | 1 |  | 1 |  |
| ≥ 65 | 3.651 [3.427-3.889] | 0.000 | 3.317[3.111-3.537] | 0.000 |
| **Tumor location** |  |  |  |  |
| Right colon | 1 |  | 1 |  |
| Left colon | 0.814[0.776-0.853] | 0.000 | 0.986[0.940-1.035] | 0.576 |
| **mStage** |  |  |  |  |
| A | 1 |  | 1 |  |
| B | 1.500 [1.397-1.611] | 0.000 | 1.368 [1.274-1.470] | 0.000 |
| C | 2.131 [1.995-2.277] | 0.000 | 1.844 [1.725-1.971] | 0.000 |
| D | 3.485 [3.180-3.819] | 0.000 | 2.892 [2.637-3.171] | 0.000 |
| E | 4.618 [4.210-5.065] | 0.000 | 3.762 [3.425-4.132] | 0.000 |
| F | 7.841 [6.649-9.247] | 0.000 | 6.761 [5.729-7.979] | 0.000 |
|  |  |  |  |  |

**Table S1 Cox regression analyses of factors related to OS in the development cohort**

| **Characteristics** | **Univariate analysis** | | **Multivariable analysis** | |
| --- | --- | --- | --- | --- |
|  | **HR [95%CI]** | **P** | **HR [95%CI]** | **P** |
| **Race** |  |  |  |  |
| White | 1 |  | 1 |  |
| Black | 1.079 [0.972-1.198] | 0.154 | 1.259 [1.133-1.398] | 0.000 |
| Other | 0.737[0.644-0.844] | 0.000 | 0.777 [0.678-0.890] | 0.000 |
| **Gender** |  |  |  |  |
| Male | 1 |  |  |  |
| Female | 1.066[0.995-1.142] | 0.070 |  |  |
| **Grade** |  |  |  |  |
| I+II | 1 |  | 1 |  |
| III+IV | 1.687[1.543-1.844] | 0.000 | 1.271 [1.161-1.391] | 0.000 |
| **Histological type** |  |  |  |  |
| Adenocarcinoma | 1 |  | 1 |  |
| Mucinous/signet-cell carcinoma | 1.191 [1.052-1.349 | 0.006 | 0.895 [0.790-1.014] | 0.082 |
| **Age (years)** |  |  |  |  |
| < 65 | 1 |  | 1 |  |
| ≥ 65 | 2.999 [2.741-3.281] | 0.000 | 2.542[2.321-2.785] | 0.000 |
| **Tumor location** |  |  |  |  |
| Right colon | 1 |  |  |  |
| Left colon | 0.952[0.886-1.023] | 0.181 |  |  |
| **mStage** |  |  |  |  |
| A | 1 |  | 1 |  |
| B | 2.178 [1.890-2.511] | 0.000 | 2.026 [1.757-2.336] | 0.000 |
| C | 4.095 [3.593-4.667] | 0.000 | 3.628 [3.182-4.138] | 0.000 |
| D | 8.422 [7.206-9.844] | 0.000 | 7.205 [6.610-8.426] | 0.000 |
| E | 13.892 [11.970-16.123] | 0.000 | 11.610 [9.985-13.498] | 0.000 |
| F | 27.564 [22.332-34.022] | 0.000 | 24.202 [19.586-29.904] | 0.000 |

**Table S2 Cox regression analyses of factors related to CSS in the development cohort (excluding died from other causes)**

**Fig. S1 Kaplan-Meier curves for patients stratified by the novel N stage in the development cohort**


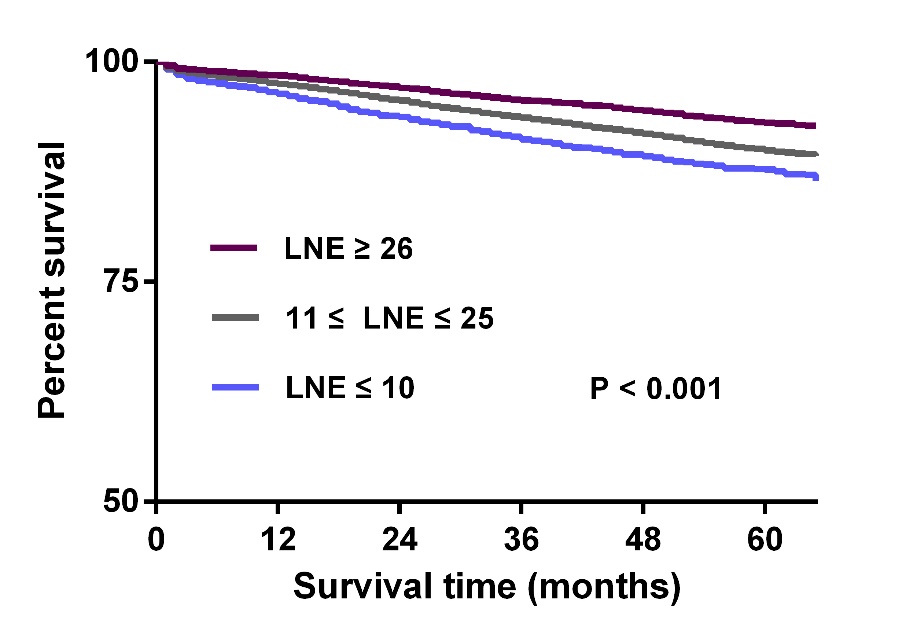

Supplement: Supplementary file 1 — Supplementary Information. [file 41598_2022_9228_MOESM1_ESM.docx]
